# Functional recovery with histomorphometric analysis of nerves and muscles after combination treatment with erythropoietin and dexamethasone in acute peripheral nerve injury

**DOI:** 10.1371/journal.pone.0238208

**Published:** 2020-09-03

**Authors:** Jung Il Lee, Jeong Min Hur, Jooyoung You, Duk Hee Lee

**Affiliations:** 1 Department of Orthopedic Surgery, Hanyang University Guri Hospital, Guri, South Korea; 2 College of Medicine, Hanyang University, Seoul, South Korea; 3 Department of Emergency Medicine, Ewha Women's University Mokdong Hospital, Seoul, South Korea; 4 College of Medicine, Ewha Women's University, Seoul, South Korea; Universitatsklinikum Wurzburg, GERMANY

## Abstract

**Introduction:**

Peripheral nerve injury (PNI) often leads to significant functional loss in patients and poses a challenge to physicians since treatment options for improving functional outcomes are limited. Recent studies suggest that erythropoietin and glucocoticoids have beneficial effects as mediators of neuro-regenerative processes. We hypothesized that combination treatment with erythropoietin and glucocoticoids would have a synergistic effect on functional outcome after PNI.

**Materials and methods:**

Sciatic nerve crush injury was simulated in ten-week-old male C57BL/6 mice. The mice were divided into four groups according to the type of drugs administered (control, erythropoietin, dexamethasone, and erythropoietin with dexamethasone). Motor functional recovery was monitored by walking track analysis at serial time points up to 28 days after injury. Morphological analysis of the nerve was performed by immunofluorescent staining for neurofilament (NF) heavy chain and myelin protein zero (P0) in cross-sectional and whole-mount nerve preparations. Additionally, morphological analysis of the muscle was performed by Hematoxylin and eosin staining.

**Results:**

Combination treatment with erythropoietin and dexamethasone significantly improved the sciatic functional index at 3, 7, 14, and 28 days after injury. Fluorescence microscopy of cross sectional nerve revealed that the combination treatment increased the ratio of P0/NF-expressing axons. Furthermore, confocal microscopy of the whole-mount nerve revealed that the combination treatment increased the fluorescence intensity of P0 expression. The cross-sectional area and minimum Feret’s diameter of the muscle fibers were significantly larger in the mice which received combination treatment than those in the controls.

**Conclusion:**

Our results demonstrated that combination treatment with erythropoietin and dexamethasone accelerates functional recovery and reduces neurogenic muscle atrophy caused by PNI in mice, which may be attributed to the preservation of myelin and Schwann cell re-myelination. These findings may provide practical therapeutic options for patients with acute PNI.

## Introduction

Acute peripheral nerve injuries (PNIs) are commonly encountered problems in patients who have experienced multiple and severe trauma. It is estimated that roughly 3% of all trauma patients experience PNI, and the annual incidence has been increasing [1, 2]. PNI frequently leads to significant functional loss and permanent physical disability, that disproportionately affects young healthy adults of working age. PNI has a very close relationship with skeletal injuries [1]. Fractures and other musculoskeletal injuries damage the adjacent nerves either by direct compression or stretch/compression by hematoma in the nerve. PNI can also occur iatrogenically during routine orthopedic surgical procedures such as compression of the nerve during fixation with screws or wires, direct insult to the nerve from soft tissue dissection, and stretching of the nerve due to soft tissue retraction. Despite the availability of modern diagnostic tools and recent microsurgical advances, patients with PNI do not attain full functional recovery [3]. Because axonal regeneration and growth support of Schwann cells diminish with increasing time and distance from the injury site, the timely regeneration of axons are required in order to regain satisfactory function [4]. Thus, promoting this regeneration process could potentially prevent negative outcomes and facilitate partial or complete functional recovery [5].

Erythropoietin, an endogenous hematopoietic cytokine, is mainly responsible for the proliferation, differentiation, and maturation of erythroid progenitor cells. Erythropoietin was the first hematopoietic growth factor to be cloned, and is one of the highest-selling biopharmaceutical products worldwide, according to the U.S. Food and Drug Administration [6]. Although the cellular and molecular-level mechanisms of PNI and its response to erythropoietin are not fully understood, recent evidence has shown that erythropoietin has therapeutic and beneficial effects on PNI. Numerous preclinical studies have shown that erythropoietin is effective in accelerating functional recovery after PNI [5, 7–15], and that it prevents axonal degeneration, protects against oxidative stress, and stimulates axonal regeneration and Schwann cell re-myelination after PNI [5, 7, 12, 13, 16, 17]. On the other hand, glucocorticoids have been widely used for treating various kinds of peripheral nerve disorders such as autoimmune neuropathy, acute spinal cord injury, traumatic optic neuropathy, facial nerve palsy, and compressive neuropathy. Glucocorticoids are often used after injury to reduce edema in the neurologic tissue and inhibit inflammatory response. It has been reported that glucocorticoids inhibit the production of nuclear factor kappa B (NF-κB) and reduce the upregulation of tumor necrosis factor-alpha, as well as transcapillary permeability [18, 19]. Moreover, previous studies showed that Schwann cells of peripheral nerves express glucocorticoid receptors, and that glucocorticoids enhance Schwann cell proliferation, myelin formation, and myelin protein zero (P0) gene expression [20–22]. Several studies also have demonstrated that glucocorticoids promote functional recovery after PNI [23–27].

One retrospective clinical study found that the pharmaceutical approach of combining erythropoietin and glucocorticoids accelerates the recovery of motor and sensory function in patients with acute postoperative nerve palsy after joint replacement arthroplasty [28]. However, it is not known whether the combination treatment has an effect on nerve regeneration, re-myelination, skeletal muscle loss, and functional recovery. We therefore designed an animal study to explore the possible effects of combination treatment with erythropoietin and glucocorticoids on functional recovery after acute sciatic nerve crush injury. Our hypothesis was that if monotherapy with erythropoietin or glucocorticoids can promote nerve regeneration and functional recovery, then combination therapy with these pharmaceutical agents should have synergistic beneficial effects.

## Materials and methods

### Mouse models of peripheral nerve crush injury

The experimental design and animal protocols were approved by the Institutional Animal Care and Use Committee (IACUC) at the Hanyang University College of Medicine (2019–0056). Ten-week-old male C57BL/6J mice weighing 20–25 g were used in this study. The mice were housed at the animal facility, and were handled according to the IACUC guidelines for the care and use of laboratory animals. The mice were anesthetized with isoflurane-mediated inhalation anesthesia. Their right hind limb and lower back were shaved, washed with 70% ethanol, and prepped with povidone iodine. A 2 cm long skin incision was made on the extended posterior right hind limb to carefully expose the right sciatic nerve through a trans-gluteal approach under an operating microscope (OPMI pico, Carl Zeiss, Oberkochen, Germany). All crush injuries were created proximal to the trifurcation site of the sciatic nerve’s sural, tibial and peroneal divisions using diagonal jawed forceps (Integra Miltex 18–1107, York, PA) for 30 seconds. Extreme care was taken to avoid inflicting additional damages to the sciatic nerve, which could affect recovery after injury. After the injury, the skin incision was closed using four or five interrupted 4–0 prolene sutures (Ethicon, Somerville, NJ). The mice were then returned to their cages, allowed to continue with free activity, and observed under the supervision of the attending veterinarian. The sutures were removed on 14 days after surgery, and functional analysis was performed at specific time points.

### Experimental design

**Fig 1:**
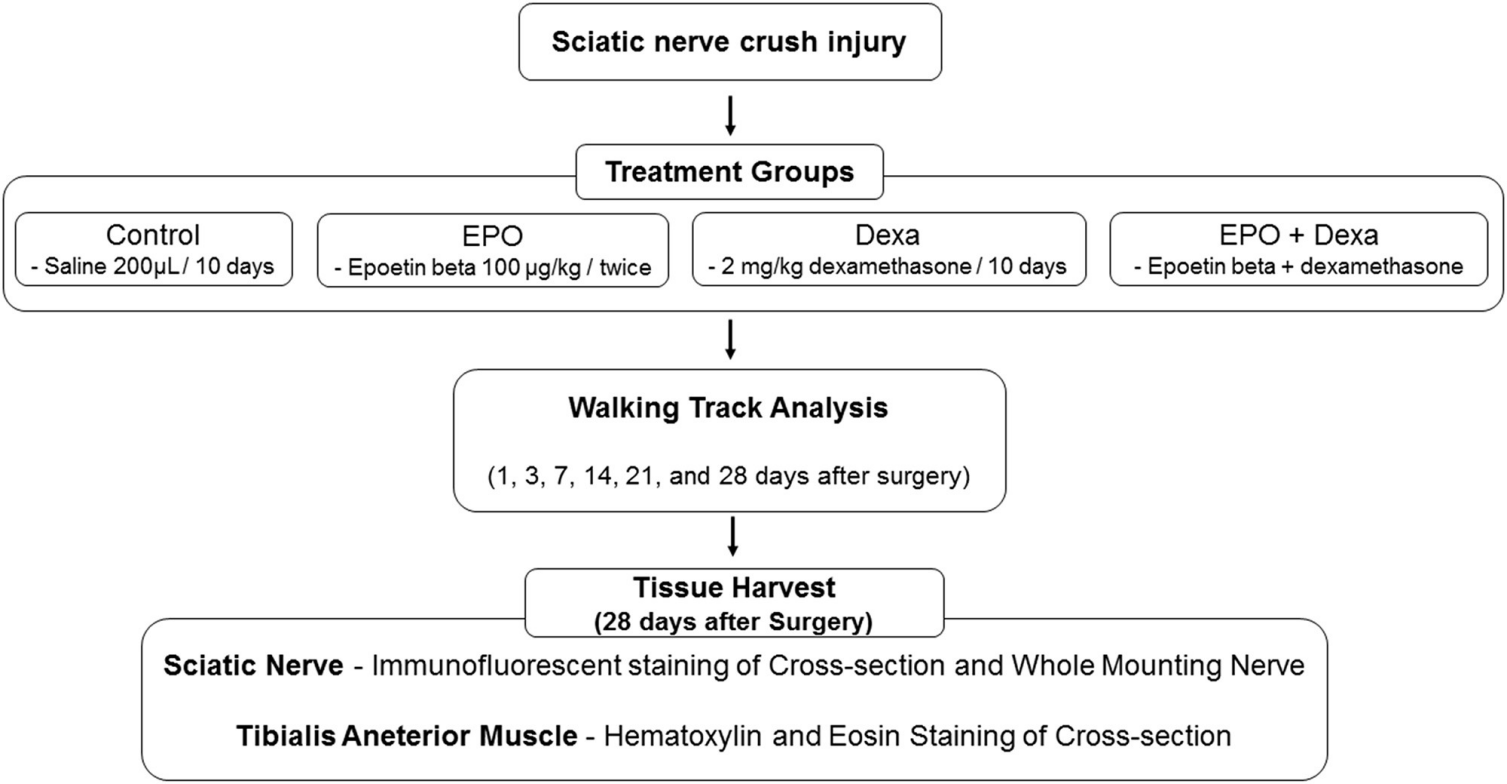
Flow chart of experimental design. Control, mice treated with saline; EPO, mice treated with erythropoietin; Dexa, mice treated with dexamethasone; EPO+Dexa, mice treated with erythropoietin and dexamethasone (n = 7 / group).

The mice were randomly assigned to four groups (n = 7/group): a control group and three treatment groups based on the different types of drugs adminstrered: erythropoietin, dexamethasone, and combination treatment (erythropoietin + dexamethasone) (Fig 1). The control mice received a daily intraperitoneal injection containing 200 μL of normal saline daily, starting immediately and up to 10 days after the crush injury. Erythropoietin-treated mice received a dose of 100 μg/kg EPO (Epoetin beta [Mircera], Roche, Basel, Swiss) every two weeks starting immediately after the crush injury. Dexamethasone-treated mice received a dose of 2 mg/kg dexamethasone (Yuhan, Seoul, South Korea) daily starting immediately and up to 10 days after the crush injury. Erythropoietin+dexamethasone-treated mice received both erythropoietin and dexamethasone. The dosages were based on previous animal studies with erythropoietin or dexamethasone. Most investigators usually used glucocorticoid (either betamethasone or dexamethasone) at a dose of 1 ~ 2 mg/Kg for 1 ~ 28 days after surgery for treating PNI in rodents [23, 24, 29]. Regarding erythropoietin, 5,000 IU/Kg was the usual dosage for treating PNI in rodents [5, 9, 12], and thus we assumed an equivalent or slightly higher dose of epoetin beta, which is a long-acting erythropoietin. Erythropoietin and dexamethasone were suspended in a 200 μL solution of sterile saline at the time of injection and delivered intraperitoneally.

We sampled 1 cc of whole blood from the mice treated with erythropoietin before injection as well as every week after injection. The blood samples were analyzed for serum hemoglobin and hematocrit to confirm the well-known hematopoietic effects of erythropoietin in these mice.

### Functional analysis by sciatic functional index

We performed a walking track analysis according to a previously described method to quantify sciatic functional index (SFI) and evaluate motor functional recovery [9, 30, 31]. Briefly, the hind paws of the mice were painted with ink, which allowed footprints to be recorded on the paper lining the floor of the walking track on days 1, 3, 7, 14, 21, and 28 after surgery. We made each mouse walk along a 70 cm-long corridor until at least three clear footprints were obtained. Two observers blinded to the study protocols selected three footprints per limb, which were measured using digital calipers. The observers recorded three parameters in both limbs (experimental limb [E] and normal limb [N]): the lengths of the third toe to heel (Print Length [PL]), the first toe to the fifth toe (toe spread [TS]), and the second toe to the fourth toe (intermediate toe spread [IT]). The SFI was calculated using the following formula described by Bain et al.: SFI = –(38.3 × (EPL-NPL))/NPL + (109.5 × (ETS-NTS))/NTS + (13.3 × (EIT-NIT))/NIT– 8.8 [30]. In general, an index of 0 indicates normal function and an index of –100 represents complete loss of function.

### Immunofluorescent staining of cross-sectioned nerves and morphometric assessments

After functional analysis on day 28 after surgery, the mice were euthanized by CO_2_ asphyxiation with cervical dislocation. The sciatic nerves were harvested, and all nerves were fixed for 5 hours in 4% paraformaldehyde at 4°C. The nerves were embedded in paraffin and cross-section were taken at the crush site. The slides were pretreated with 0.01 M citrate buffer (pH 6.0) for antigen retrieval. Nonspecific blocking was performed with 1:20 diluted goat serum (ab7481, Abcam, Cambridge, UK). Sequential sectioned slides were incubated with anti-P0 myelin antibody (P0, 1:200, ab31851; Abcam) and anti-neurofilament (NF) heavy chain antibody (1:500, ab4680; Abcam) separately in 5% BSA PTX (phosphate-buffered saline [PBS] with 1% Triton X-100 [Sigma X100]). Incubation with 4′,6-diamidino-2-phenylindole (DAPI 1:1000) and fluorescent secondary antibodies (Alexa Fluor 488, and 594-conjugated antibodies [1:500, ab197485 and ab150088, Abcam)) was performed after washing the sections in buffer to remove the primary antibodies. Fluorescent images of cross-sectioned nerves were captured using a Leica DMI4000B fluorescence microscope (Leica microsystems, Wetzlar, Germany).

Three cross-sectioned nerve images per group were used to evaluate and quantify the NF- and P0-labeled axons. Images were analyzed in a semiautomatic fashion using ImageJ (National Institutes of Health, Bethesda, MD) to determine the number of NF or P0-labeled axons.

### Immunofluorescent staining of whole-mount nerves

Whole-mount immunofluorescent staining of nerves was performed according to a previously described method to evaluate axonal regeneration and re-myelination [32]. Briefly, after SFI analysis 28 days after surgery, the nerves were collected and fixed for 5 hours in 4% paraformaldehyde at 4°C. The nerves were then washed three times for 10 minutes each with PTX and incubated in a blocking solution (10% normal goat serum in 5% BSA PTX) overnight at 4°C. The next day, the nerves were transferred into two primary antibodies (NF, 1:1000; P0, 1:500) in 5% BSA PTX and incubated for 72 hours at 4°C with gentle rocking. The nerves were then washed with PTX for 4 hours at 4°C, with a change of PTX every 1 hour. Thereafter, the nerves were incubated with DAPI (1:1000), Alexa Fluor 488, and 594-conjugated secondary antibodies (1:500) for 48 hours at 4°C with gentle rocking. The nerves were washed in PTX three times for 15 minutes each, followed by 4 hours of washing in PTX with a PTX change every hour. The nerves were then left overnight at 4°C without changing the PTX. The next day, the nerves were washed with PBS three times for 10 minutes to remove the triton, and were cleared sequentially in 25% and, 50% glycerol (Sigma G6279) in PBS for 6 and 12 hours, respectively. After clearing, the nerves were mounted in Anti-Fade Fluorescence Mounting Medium (ab104135, Abcam). Stained whole nerves were imaged using a Leica DMI4000B fluorescence microscope (Leica microsystems, Wetzlar, Germany). Six individual images were combined into one image using Adobe Photoshop software (Adobe Systems) to obtain the whole nerve image. The crush sites of the stained nerves were captured using a Leica TCS SP5 confocal microscope in order to assess the regeneration of the axon and myelin.

### Morphometric assessments of whole-mount nerves

Four confocal nerve images at the crush site per group were analyzed to evaluate the number of NF expressing axons as well as the fluorescence intensity (F.I) of P0-expressing myelin. Quantitative analysis of confocal images was carried out automatically using the Leica Application Suite X (LAS X) software, which provides automated measures of F.I. of the selected area. The graphs menu in LAS X show histograms that have been measured a gray-scale values ranging from pure black (0) to pure white (255) from the linear regions of interest. The number of bars in the histograms as well as the mean of the gray-scale values of the linear regions of interest were also calculated. The perpendicular lines of the nerve fibers were drawn on the nerve images distal to the crush site at 500, 1000, and 1500 μm. The histograms according to F.I on three perpendicular lines were generated and the mean values of F.I on the three lines were also acquired. The numbers of NF-expressing axons distal to the crush site at 500, 1000, and 1500 μm were measured by the number of histogram bars on three lines in the NF-stained images, and the F.I of P0-expressing myelin was measured distal to the crush site at 500, 1000, and 1500 μm by the mean value on these lines in the P0-stained images.

### Hematoxylin and eosin staining and morphometric assessments of muscles

The tibialis anterior (TA) muscles of the injured hind limb were harvested from the surrounding tissues 28 days after surgery. The harvested TA muscles were immersed in 4% paraformaldehyde for histological examination. They were then embedded in paraffin and cross-sectioned longitudinally at 5 μm, stained with hematoxylin and eosin, and imaged using a BX53 microscope with a DP72 camera (Olympus USA, Center Valley, PA). Quantitative analysis for the cross-sectional area (CSA) and minimum Feret’s diameter (MFD) was carried out using Image J software. Three random microscopic fields were chosen from each muscle and three animals were analyzed per group.

### Statistical analysis

All results are presented as means ± standard error of mean. Statistical analysis was performed using the Student t-test and one-way ANOVA-followed by Tukey’s post-hoc test for multiple comparisons.

## Results

### Combination treatment with erythropoietin and dexamethasone accelerates functional recovery

Firstly, we obtained the blood cell counts of the mice treated with erythropoietin to confirm the hematopoietic effect of erythropoietin. Hemoglobin and hematocrit levels in the mice treated with erythropoietin 7 days after the injection, were significantly higher than those in the mice treated with saline (S1 Fig).

**Fig 2:**
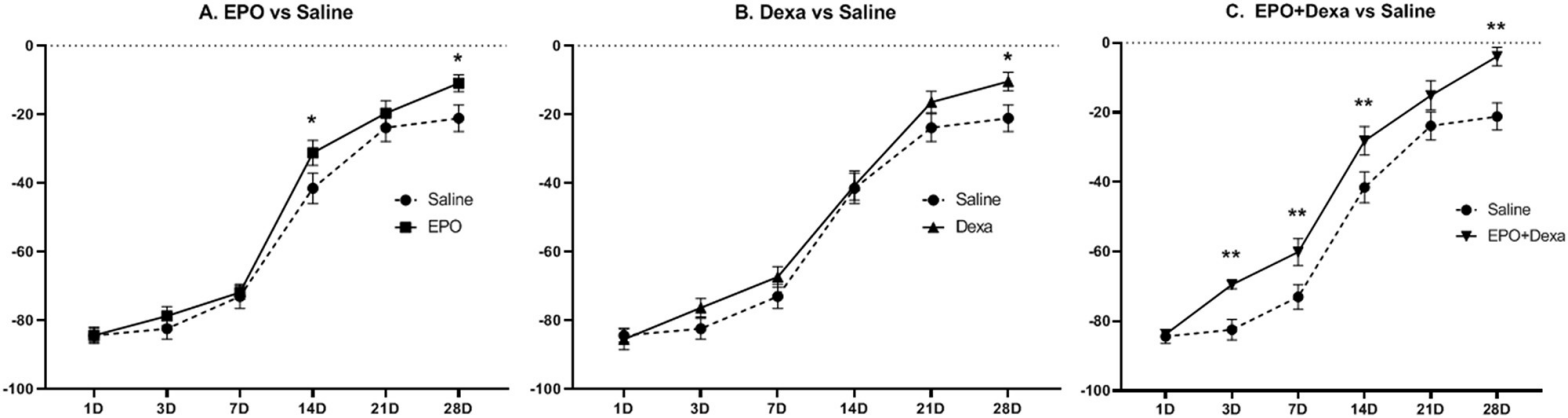
Functional improvement after combination treatment with erythropoietin and dexamethasone for mouse sciatic nerve crush injury. (A and B) Hind limb motor function as indicated by the sciatic functional index (SFI) in mice treated with erythropoietin or dexamethasone was better than that in control mice treated with saline at various time points. However, it was significantly higher only at 28 days after injury. (C) SFI of mice treated with combination therapy was significantly higher than that of control mice at various time-points except at 21 days after surgery, suggesting accelerated functional recovery via synergistic effects. (n = 7/group; *P<0.1, **P<0.05; saline, mice treated with saline; EPO, mice treated with erythropoietin; Dexa, mice treated with dexamethasone; EPO+Dexa, mice treated with erythropoietin and dexamethasone).

Since restoration of motor function is the primary goal of PNI treatment, we first examined the effect of the drugs on the functional outcomes. Overall, all mice subjected to the crush injury lost sciatic nerve function on the first day after injury. Sciatic nerve function, as measured by the SFI, recovered gradually over the 28 days after injury (Fig 2). The mice treated with erythropoietin, dexamethasone, and their combination had significantly better SFI than the mice treated with saline at 28 days after injury (Saline, -21.1 ± 3.9; erythropoietin, -10.9 ± 2.5; dexamethasone, -10.4 ± 2.7; erythropoietin+dexamethasone, -3.9 ± 2.7). However, the mice treated with erythropoietin+dexamethasone showed better SFI than the control mice at 3, 7, and 14 days after injury.

### Combination treatment increases re-myelination

**Fig 3:**
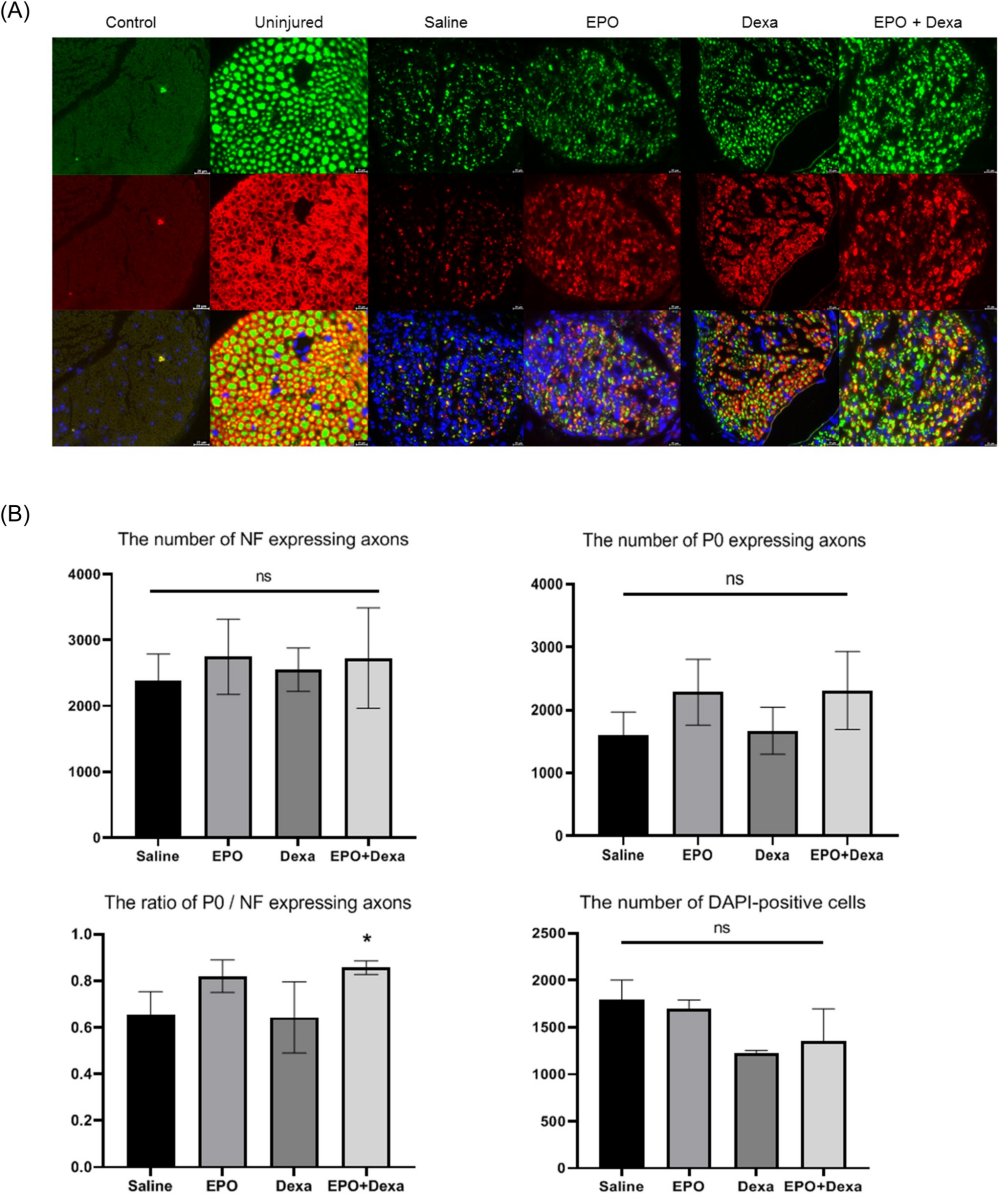
Preservation of myelinated axons after combination treatment with erythropoietin and dexamethasone for mouse sciatic nerve crush injury. (A) Representative cross-sectional images of the nerve at the crush site upon immunofluorescence staining (× 200). Combination treatment increased the number/size of axons and myelin compared with the saline treatment. (B) Combination treatment did not increase the number of NF or P0-expressing axons, but increased the ratio of P0 / NF expressing axons. (n = 3/group; *P < 0.1 vs. saline-treated group; green (Alexa Fluor 488 1:500, ab197485, Abcam), neurofilament (NF) heavy chain (1:500, ab4680, Abcam); red (Alexa Fluor 594 1:500, ab150088, Abcam), myelin protein 0 (P0, 1:200, ab31851, Abcam); blue (4′,6-diamidino-2-phenylindole [DAPI] 1:1000, ab228549, Abcam); control, staining without primary antibody; uninjured, normal nerve; saline, nerve treated with saline; EPO, nerve treated with erythropoietin; Dexa, nerve treated with dexamethasone; EPO+Dexa, nerve treated with erythropoietin and dexamethasone).

To determine the functional benefit of drug therapy, nerves of the mice were harvested 28 days after injury and analyzed both qualitatively and quantitatively through immunofluorescence staining of cross-sectional and whole-mount nerve preparations. Antibodies to NF and P0 were used to evaluate the underlying changes in axonal continuity and myelination status, respectively. A cross-sectional image of an uninjured nerve (Fig 3A) showed large homogenous axons enveloped with thick round myelin sheath. All images of crushed nerves (Fig 3A) showed decreased numbers/sizes of axons as well as loss of myelin. However, nerves that were subjected to combination treatment (Fig 3A) showed increased numbers/sizes of axons and myelin than the nerves of control mice. Dexamethasone and combination treatment showed only a few blue cells in the merged images, suggesting that dexamethasone reduced inflammatory responses. However, there were no statistically significant differences in the number of DAPI-positive cells. There were no significant differences in the number of NF or P0-stained axons among the four treatment groups. However, combination treatment increased the ratio of P0/NF-expressing axons (Fig 3B; saline, 0.65 ± 0.06; erythropoietin, 0.82 ± 0.04, dexamethasone, 0.64 ± 0.09; erythropoietin + dexamethasone, 0.86 ± 0.02).

**Fig 4:**
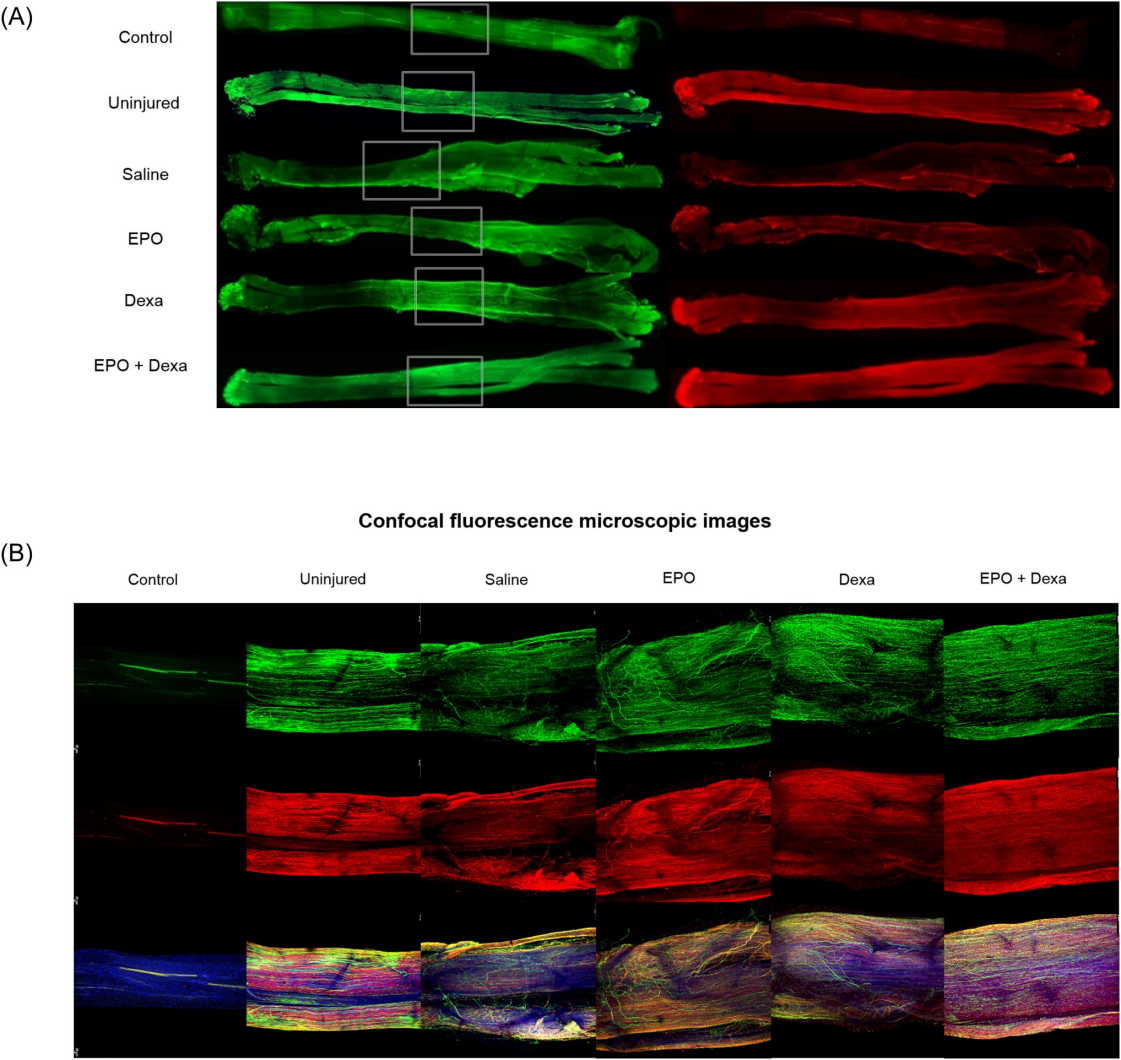
Representative whole-mount nerve images (A) and confocal images of crush injury site of whole-mount nerves (B) from different groups. Uninjured nerves show normal nerve morphology with unidirectional NF-stained axons aligned in parallel as well as homogenously P0-stained myelin. All images of crushed nerves showed that the NF-stained crushed axons re-grew multidirectionally to regenerate the distal side. All pharmacologic treatments increased the number of NF-stained axons and the fluorescence density of P0-stained myelin compared to the saline treatment. These findings are evident from the confocal images of crushed nerves treated with both erythropoietin and dexamethasone. (n = 4 / group; green (Alexa Fluor 488 1:500, ab197485, Abcam), neurofilament (NF) heavy chain (1:500, ab4680, Abcam); red (Alexa Fluor 594 1:500, ab150088, Abcam), myelin protein 0 (P0, 1:200, ab31851, Abcam); blue (4′,6-diamidino-2-phenylindole [DAPI] 1:1000, ab228549, Abcam), the proximal side is on the left, and the distal side is on the right.; control, staining without primary antibody; uninjured, normal nerve; Saline, mice treated with saline; EPO, mice treated with erythropoietin; Dexa, mice treated with dexamethasone; EPO+Dexa, mice treated with erythropoietin and dexamethasone).

**Fig 5:**
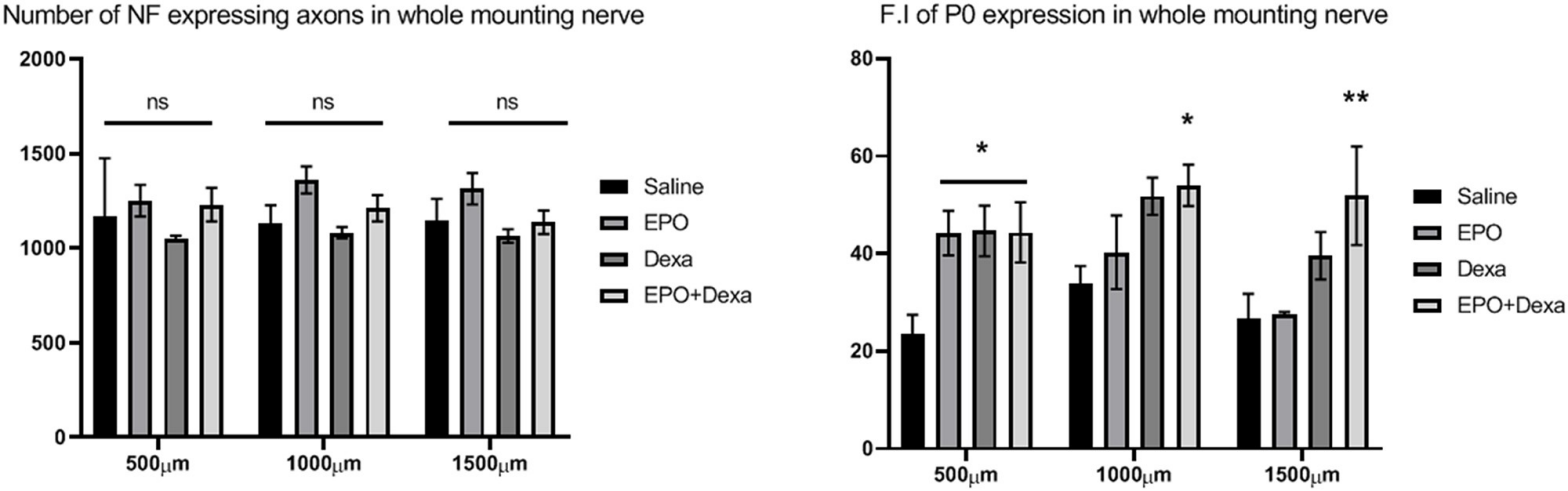
Increased fluorescence intensity (F.I) of myelin after the combination treatment with erythropoietin and dexamethasone for mouse sciatic nerve crush injury. (A) The numbers of axons at different distances in the erythropoietin and erythropoietin + dexamethasone treatment groups were larger those that in the control (saline treatment) groups, but these differences were not significant. (B) The F.I of myelin at different distances in the pharmacological treatment groups was larger than that in the saline-treated group. These findings were significant at a distance of 500 μm for all the pharmacological treatment groups and at 1000 and 1500 μm for the erythropoietin+ dexamethasone treatment group. (n = 4 / group; *P < 0.1, **P < 0.05 vs. saline-treated group; F.I, fluorescence intensity; saline, mice treated with saline; EPO, mice treated with erythropoietin; Dexa, mice treated with dexamethasone; EPO+Dexa, mice treated with erythropoietin and dexamethasone).

Whole-mount nerve images were captures using both a fluorescence (Fig 4A) and confocal microscope (Fig 4B) to visualize any differences in axonal continuity and myelination status. Uninjured control nerves showed normal architecture with uniform NF and P0 staining and unidirectional nerve fibers being aligned in parallel. All injured nerves after crush injury displayed misdirected nerve fibers, which were least prominent in the nerve treated with the combination treatment, as well aslow and heterogeneous signal intensity of NF and P0 staining compared to the uninjured nerve. The number of NF-stained axons in the treated nerves was not different from that in the saline-treated control nerve. P0 signal intensity of the nerve treated with combination treatment was more pronounced than that of the nerve treated with saline, erythropoietin, or dexamethasone. Combination treatment led to a significant increase in the F.I of P0 expression of nerves at 500, 1000 and 1500 μm distal from the crush site compared with saline treatment (Fig 5; 500 μm- Saline, 22.5 ± 4.1; erythropoietin+dexamethasone, 44.4 ± 6.2; 1000 μm- Saline, 33.4 ± 4.1; erythropoietin+dexamethasone, 54 ± 4.2; 1500 μm- Saline, 27.6 ± 5.2; erythropoietin+dexamethasone, 51.9 ± 10.1).

### Combination treatment attenuates denervation-induced muscular atrophy

**Fig 6:**
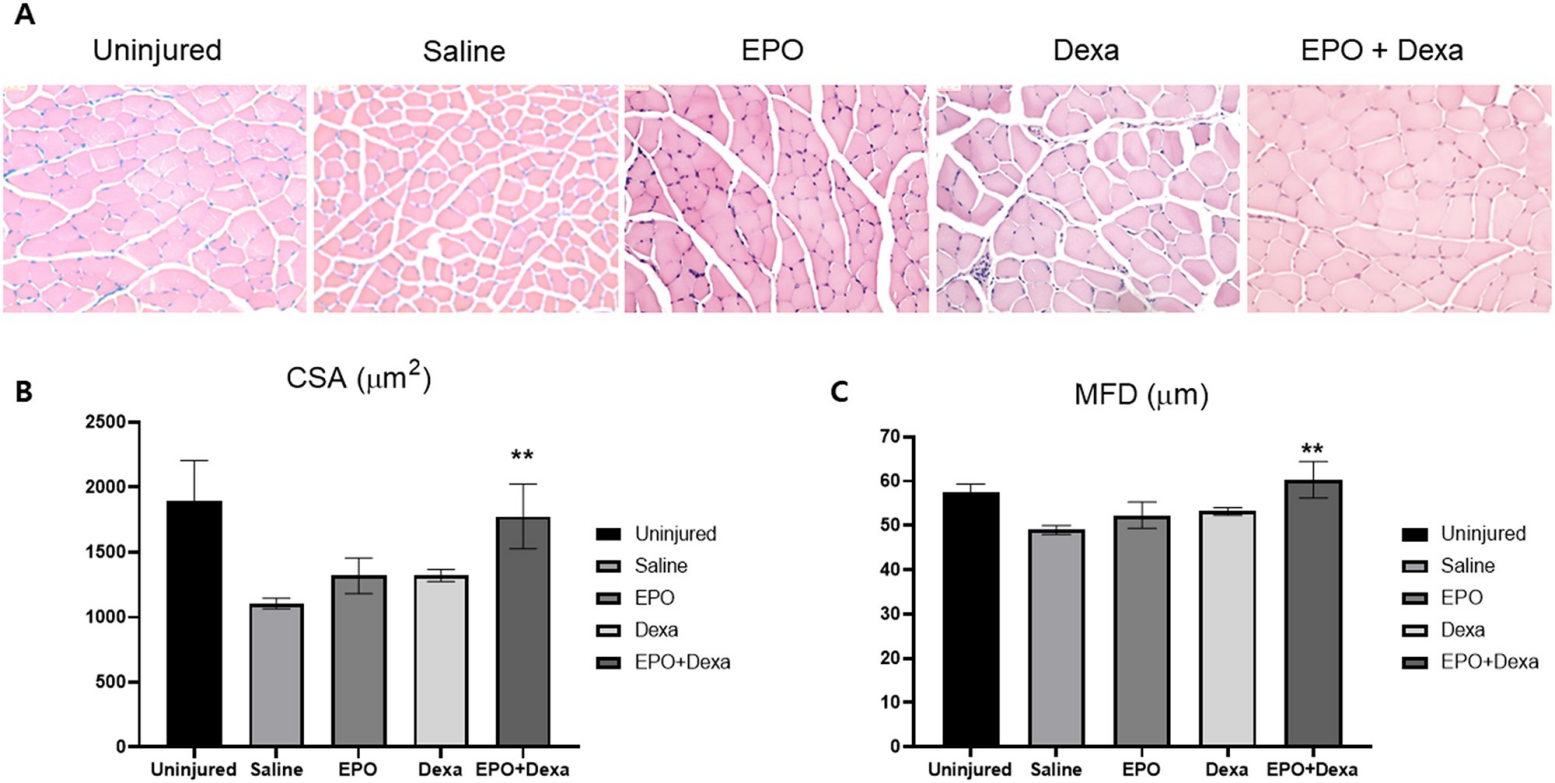
Reduction in denervation-induced atrophy of muscle fibers after combination treatment with erythropoietin and dexamethasone. (A) Representative H & E images of transverse sections of the tibialis anterior (TA) muscle from different groups (× 200). The saline-treated group showed decreased muscle fiber, increased intermyofiber spacing and fibrotic changes compared to the uninjured group. Pharmacologic treatments reversed these effects, which was most signigicant in the erythropoietin+dexamethasone group. (B and C) Crush injury of the sciatic nerve reduced the cross-sectional area (CSA) and minimum Feret’s diameter (MFD) of the muscle fiber, but the pharmacological treatments allowed the atrophied muscle fibers to recover. In particular, the erythropoietin+dexamethasone treatment group showed significantly larger CSA and MFD than the saline-treated group, indicating minimum muscle atrophy. (n = 3 / group; **P < 0.05 vs. saline-treated group; saline, mice treated with saline; EPO, mice treated with erythropoietin; Dexa, mice treated with dexamethasone; EPO+Dexa, mice treated with erythropoietin and dexamethasone).

Quantitative analysis for CSA and MFD of the muscle fiber was performed with Image J software to evaluate the degree of muscular atrophy caused by nerve crush injury (Fig 6). Measurements of the CSA and MFD revealed extensive muscle atrophy in the injured mice treated with saline compared to that in the uninjured mice (uninjured: 1890.8 ± 314.6 μm2, 57.5 ± 1.9 μm; saline: 1105.4 ± 39.9 μm2, 48.5 ± 1 μm). All pharmacologic treatments, but mot the saline treatment, protected the CSA and MFD of the muscle fiber, and this protective effect was found to be highest with the combination treatment (erythropoietin, 1318.8 ± 137.1 μm^2^, 52.5 ± 3 μm; dexamethasone, 1321 ± 45.7 μm^2^, 53 ± 0.9 μm; erythropoietin +dexamethasone, 1775.5 ± 247.6 μm^2^, 60 ± 4.1 μm).

## Discussion

**Fig 7:**
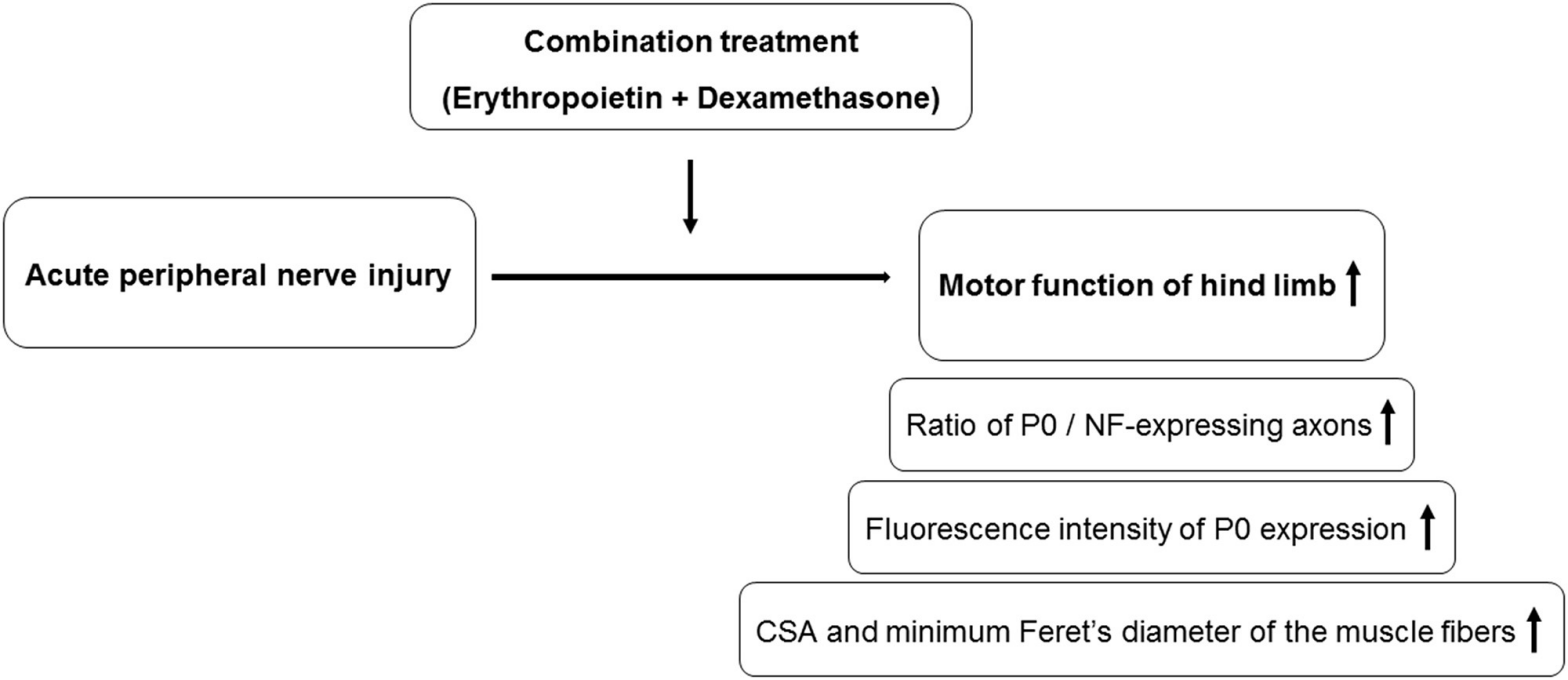
Schematic summary of effect of combination treatment.

We report the synergistic effects of combination treatment with erythropoietin and dexamethasone on re-myelination, denervation-induced muscle atrophy, and global motor function after sciatic crush injury in a mouse model (Fig 7). The present study’s results demonstrate that the combination treatment accelerated motor functional recovery, as measured by SFI in as early as day 3 after therapy initiation, possibly by promoting Schwann cell re-myelination, as per histomorphometric analysis. We demonstrate that, in addition to accelerating functional recovery, combination treatment also reduces denervation-induced muscle atrophy by promoting an increase in muscle fiber size.

Because our primary goal was to show functional recovery with combination pharmacotherapy, we chose to use an established murine model of sciatic nerve crushing injury [3, 5, 9, 11, 24]. Evaluating the functional response of animals with PNI to drug treatments is critical in evaluating their potential for actual clinical use in human. Histomorphometric measurements of nerve regeneration (the number of regenerated axons and myelinated axons and muscle fiber size, etc.) and assessment of cellular mechanisms of pharmacotherapy are also important for determining the process of functional regeneration after PNI. However, improvements in histomorphometric measurements are not synonymous with improvement in function. Further histologic, cellular, molecular, and genetic studies may be considered if the candidate agent leads to successful functional outcomes in animal models. The rodent sciatic nerve-crush model is a well-established model in both rats and mice, and allows for performing functional assays for motor nerve regeneration [9].

Previous studies suggest that glucocorticoids have a positive effect on functional recovery in PNI [23–27]. Al-Bishri et al. found that systemic administration of betamethasone improved functional recovery as measured by SFI, after sciatic nerve crush injury in rats [23]. Systemic administration of dexamethasone promotes functional recovery in rats after sciatic nerve crush injury, as evaluated by walking track analysis [24, 27]. The functional benefits of topical administration of betamethasone or dexamethasone were also reported in a study wherein these treatments were applied on the gap between transected nerve ends treated with a conduit graft [25, 26]. Evidence from preclinical studies on spinal cord injury suggest that glucocorticoids attenuate nerve damages by reducing proinflammatory cytokine production and tissue edema [33], lipid peroxidation, inducible nitric oxide synthase activity, and neuronal apoptosis [34] as well as by stabilizing calcium and sodium transcellular flux [35].

Glucocorticoids exhibit their neuroprotective effect against PNI by alleviating the consequences of neural inflammatory responses mediated by inflammatory cytokines and enzymes such as NF-κB and phospholipase A2, which lead to subsequent neutrophil, macrophage and lymphocyte recruitment [18, 24, 36]. It has been documented that glucocorticoids do not only mitigate neural inflammation but also promote Schwann cell re-myelination following PNI [24, 26, 37]. Glucocorticoid receptors (GRs) are expressed in cultured Schwann cells of the peripheral nerve [38, 39]. Glucocorticoids enhance Schwann cell proliferation and the rate of myelin formation, which are mediated through GRs [20, 21, 40]. Glucocorticoids stimulate the activity of PMP22 and P0 gene promoters [22]. Glucocorticoids have a myeloprotective effect against demyelination and an augmentative effect on re-myelination after PNI [37].

The functional benefits of erythropoietin in treating acute PNI have been reported [5, 7–15]. Elfar et al. demonstrated that erythropoietin improves motor functional recovery, as measured by SFI, after acute sciatic nerve crush injury in mice, possibly due to upregulation of the erythropoietin receptor (EPOR) in the nerve tissues after erythropoietin treatment [9]. The beneficial effects of erythropoietin on pain behavior, nerve recovery, motor strength, and muscle restoration have also been found in rats who underwent combined muscle-nerve crush injury [10]. Functional benefit after administration of erythropoietin were also found when using the rodent model of end-to-side neurorrhaphy after complete transection of the sciatic nerve [8] and in the rodent model of a conduit graft which induced a segmental defect of the sciatic nerve [11]. Previous studies suggest that these benefit may, in part, be attributable to the ability of erythropoietin to inhibit both apoptosis and the reactive increases in inflammatory cytokines [7, 41], promote mobilization of endothelial progenitor cells and angiogenesis [42], protect against oxidative damages [43], and stimulate axonal regeneration and Schwann cell re-myelination [5, 12, 44].

EPORs are present in a wide variety of non-erythroid cells throughout the body and may impact many biological functions [6, 44]. EPORs are expressed in the central nervous system and on the myelin sheath of radicular nerves in humans [45, 46]. The presence and upregulation of EPOR in the Schwann cells surrounding an injured sciatic nerve was observed in rodents [7, 47]. Although the mechanism of erythropoietin in peripheral nerve regeneration is poorly understood, it is thought to exert its effect by inducing the expression of myelin genes, peripheral myelin protein-22 (PMP22) and P-0 Myelin protein (P0), through the EPORs [48]. In vitro, erythropoietin treatment preserves myelin and promotes myelin formation in co-cultures of Schwann cells and dorsal root ganglion neurons [5]. In vivo, erythropoietin was also found to increase the ratio of P0 to NF in cross-sections of injured sites [5, 12]. Erythropoietin has a positive effect on recovery after PNI via its ability to preserve and/or promote Schwann cell re-myelination [5, 12, 44]. In addition to its myeloprotective effects, erythropoietin also has neuroprotective effects which prevents axonal degeneration as well as promotes neurite sprouting and regrowth to restore axonal continuity [17, 49, 50].

Our results show that erythropoietin and dexamethasone promote motor functional recovery, as per the walking track analysis, after PNI. Mice treated with a single agent (erythropoietin or dexamethasone) had higher SFI than the control mice at all-time points, but this difference was statistically significant only at 28 days after injury. In previous murine study, significant SFI improvements during the earliest time points after erythropoietin treatment were only observed in not mild or severe but moderate crushed sciatic nerve injury. Thus, it was assumed that injury severity mediates the beneficial effects that erythropoietin may have on functional recovery [12]. Previous studies also found no improvement in motor functional recovery in crush injured sciatic nerves in rodents two weeks after glucocorticoids treatment, with the beneficial effect of glucocorticoids only observed three weeks after treatment [23, 24]. Our SFI results show that combination treatment with erythropoietin and dexamethasone improves motor functional recovery as early as 3 and up to 28 days after PNI, which differ from the results obtained after treatment with single agents. This finding suggests that combination treatment with erythropoietin and dexamethasone is a viable therapeutic option that can be used in the clinical setting in order to optimize the posttraumatic course of patients with acute PNI. This is because nerve recovery must outstrip the degradation of the denervated motor end plate [5, 51]. Hoping that when both drugs are combined, their protective effects might be synergistic, a few clinical studies have attempted to investigate if there would be improvements in functional recovery after PNI or peripheral neuropathy using combination treatment. Combination treatment with erythropoietin and tapered oral steroids for patients suffering from iatrogenic nerve injuries after total joint replacement arthroplasty demonstrated a faster and more complete recovery of motor and sensory function compared to those reported in previous clinical studies [28]. Combination treatment with erythropoietin and high doses of steroids for patients with toxic optic neuropathy led to structural and functional improvements [52, 53].

Although these clinical studies and our functional data found a synergistic effect of both drugs on peripheral nerves, one animal study modeling contusive spinal cord injury showed that coadministration of glucocorticoid antagonized the beneficial effects of erythropoietin [54]. They speculated several possible mechanisms for this antagonistic effect, such as decreased EPOR, glucocorticoid-induced vascular dysfunction, and increased clearance of erythropoietin, but there were no supporting data provided regarding the possible underlying mechanisms. In contrast, another animal study modeling optic neuritis found a synergistic effect with combination treatment using both drugs [55]. The synergistic effect with the use of both drugs may be explained by the ability of erythropoietin to completely abolish the negative effect of glucocorticoids on neuronal survival by antagonistic regulation of the phosphorylation of mitogen-activated protein kinases. Our histomorphometric analysis revealed that combination treatment increased the ratio of P0 / NF-expressing axons in the cross-sections of the crush site as well as P0-expressing myelin in whole-mounted nerves. These findings suggest that the functional benefits of the combination treatment may be mediated by their preservation of myelin and/or promotion of Schwann cell re-myelination in treated nerves. The anti-inflammatory effect of glucocorticoids may have a positive influence on functional recovery by preserving the injured axon or a negative influence on recovery by reducing clearance of myelin debris by macrophages during Wallerian degeneration [26, 56]. We speculate that combination treatment with erythropoietin and glucocorticoids allows minimization of the negative anti-inflammatory effects, associated with the use of glucocorticoids while enhancing its myeloprotective effects.

Our study has some limitations. First, we evaluated SFI based on a walking track analysis. Although SFI assessment is the gold standard for evaluation of functional recovery after sciatic nerve injury, it tends to have a bias toward the best possible gait that the mouse can perform [12, 30, 31]. The mice were allowed to walk down the corridor three times or more per trial, giving them the best opportunity to walk as normally as they could. Second, the number and the F.I of P0 expression were investigated to confirm re-myelination after PNI, but these do not fully reflect the functional status of myelin. Electron microscopy would be helpful in assessing the functional status of the myelin sheath encircling the single axon. Third, we did not perform molecular and cellular studies to investigate the mechanisms of action of the combination treatment in nerve and muscle regeneration after PNI.

## Conclusions

Taken together, our data suggest that combination treatment with erythropoietin and dexamethasone accelerates functional recovery and reduces neurogenic muscle atrophy after nerve-crushing injury, which may be attributed to preservation of myelin and Schwann cell re-myelination. These findings show that combination treatment with erythropoietin and dexamethasone may be a practical therapeutic options for patients with acute PNI.

## Supporting information

S1 Fig Confirmation of the hematopoietic effects of erythropoietin administration in mice. (A) Serum hemoglobin increased by about 4–5 g/dL three weeks after initial administration of erythropoietin. Serum hemoglobin levels in saline-injected mice did not increase. (B) Serum hematocrit percentages of mice treated with erythropoietin were also higher than those of control mice. (n = 4/group; *P < 0.01; saline, mice treated with saline; EPO, mice treated with erythropoietin).
(TIF)

S1 Data EPO, dexa project. (PZFX)
