# Supplementary figures and images for: Functional recovery with histomorphometric analysis of nerves and muscles after combination treatment with erythropoietin and dexamethasone in acute peripheral nerve injury
Source: PLoS One. 2020 Sep 3;15(9):e0238208. doi: 10.1371/journal.pone.0238208 (PMC7470391; doi:10.1371/journal.pone.0238208)

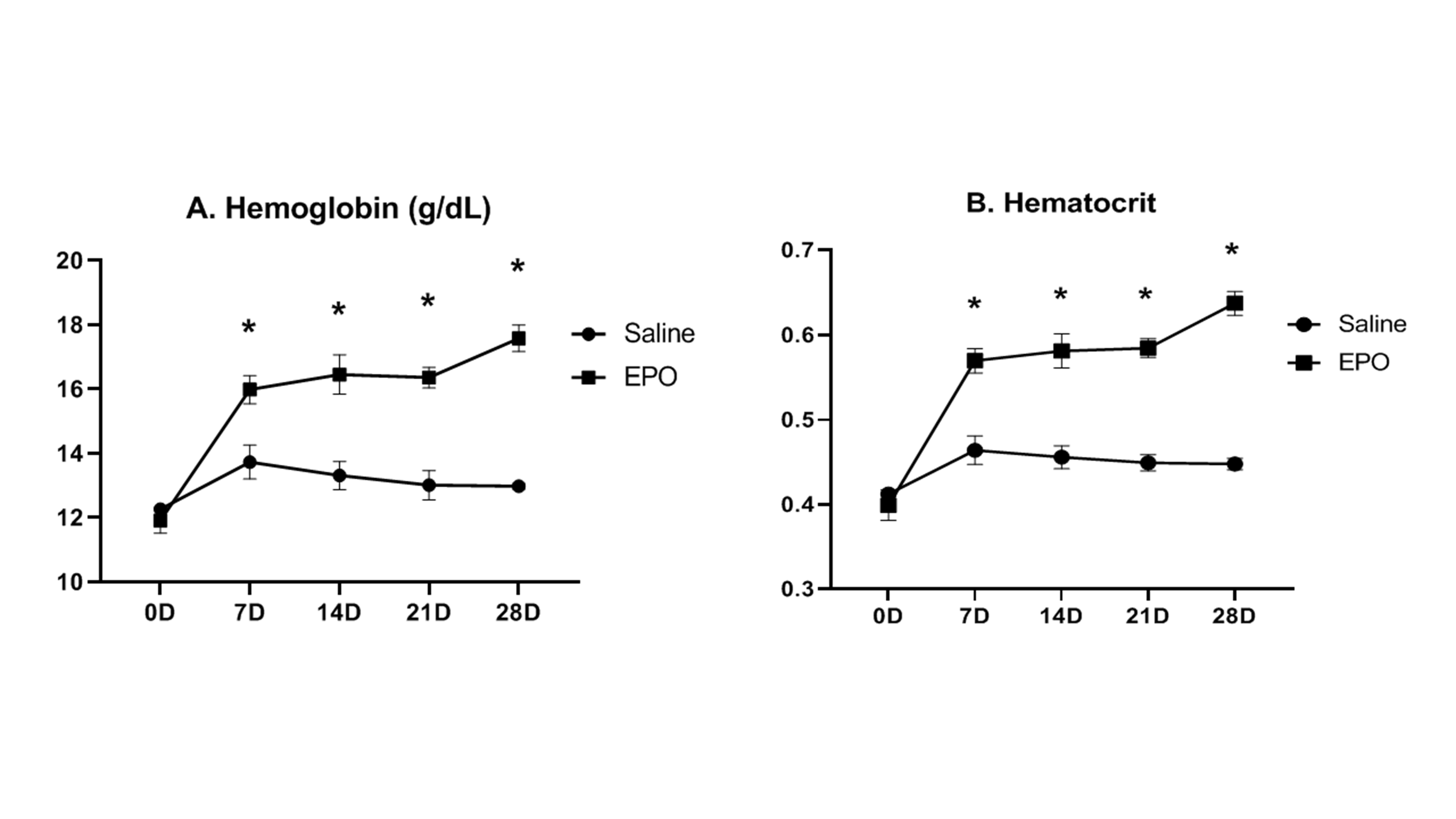

Supplement: S1 Fig — (A) Serum hemoglobin increased by about 4–5 g/dL three weeks after initial administration of erythropoietin. Serum hemoglobin levels in saline-injected mice did not increase. (B) Serum hematocrit percentages of mice treated with erythropoietin were also higher than those of control mice. (n = 4/group; *P < 0.01; saline, mice treated with saline; EPO, mice treated with erythropoietin). (TIF) [file pone.0238208.s001.tif]
